# Effectiveness of Injected Platelet-Rich Plasma in the Treatment of Diabetic Foot Ulcer Disease

**DOI:** 10.7759/cureus.28292

**Published:** 2022-08-23

**Authors:** Asad Ullah, Syed I Jawaid, Pir Naveed Ahmed Ahsan Qureshi, Tehreem Siddiqui, Khadija Nasim, Kantash Kumar, Shafqat Ullah, Mustafa Sajjad Cheema, Nikita Kumari, Hafiza Azad Elias

**Affiliations:** 1 Department of Diabetes and Endocrinology, Medical Teaching Institution (MTI) Lady Reading Hospital, Peshawar, PAK; 2 Department of Medicine, Liaquat College of Medicine and Dentistry, Karachi, PAK; 3 Department of Plastic and Reconstructive Surgery, Liaquat University of Medical and Health Sciences, Hyderabad, PAK; 4 Department of Medicine, Peoples University of Medical and Health Sciences for Women, Nawabshah, PAK; 5 Department of Medicine, Imam Clinic, Karachi, PAK; 6 Department of Medicine, Dow University of Health Sciences, Civil Hospital Karachi, Karachi, PAK; 7 Department of Medicine, Bahawal Victoria Hospital, Bahawalpur, PAK; 8 Department of Medicine, Combined Military Hospitals (CMH) Lahore Medical College, Lahore, PAK; 9 Department of Medicine, Jinnah Sindh Medical University, Karachi, PAK; 10 Department of Medicine, Teklehaimanot General Hospital, Addis Ababa, ETH

**Keywords:** growth factors, healing outcomes, conventional dressing, epithelialization, ulcer, diabetic foot, platelet rich plasma, efficacy

## Abstract

Objectives

Platelet-rich plasma (PRP) has become quite a popular course of treatment and has tremendous healing properties. Our research question inquired about the effectiveness of injected formula of PRP as the cure for diabetic foot ulcer in comparison to the conventional dressing.

Methodology

A prospective observational study was conducted at the Department of Diabetes and Endocrinology, Lady Reading Hospital, Peshawar, Pakistan, between July 2020 to January 2021. Patients' data were collected from the department's database after taking approval from the department. In our study, the selected patients were categorized into two equal groups - i.e. 80 cases in each group and were randomized by using randomization allocation software. In group I (study group) patients received PRP (1 ml /1 cm^2^) around the wound edges and in the base of the ulcer, while group II (control group) patients were treated with conventional dressing. Each patient was inspected for wounds on days 0, 14, 28, 90, and 180 on the basis of Wagner's classification of wounds to assess efficacy. A proforma was used to collect the required data and then utilized electronically for research analysis.

Results

The mean ± SD of age was 54.4±8.56 and 57.7±10.1 years in the injected PRP (study) and conventional dressing (control) groups, respectively. Of the 30 patients, 13 (43.3%) males and 17 (56.7%) females were enrolled in the study group, while 14 (46.7%) males and 16 (53.3%) females were included in the control group. The PRP was found effective in reducing the wound in about 64 (80%) patients, while wound dressing was effective in 37 (46.25%) patients (p<0.0001). In female patients, the wound healing was significantly better in the study group as compared to the control group (p<0.0001). Moreover, in patients aged above 55 years, frequently higher rates of wound reduction were observed in the study group as compared to the control group (p<0.0001).

Conclusion

The study concluded that injected PRP was significantly better than conventional dressing in the management of diabetic foot ulcer. More clinical trials are necessary to evaluate the effectiveness of injected PRP to validate the current findings.

## Introduction

Diabetes-related foot ulcers are a significant medical issue. In people with diabetes mellitus, complications from foot ulcers are the main reason for hospitalizations and amputation. More than 80% of non-traumatic amputations are caused by chronic diabetic foot ulcers, which also account for approximately 50% of foot ulcer hospital admissions each year [1]. This incidence has major clinical and financial consequences. According to data from the National Hospital Discharge Survey, United States, diabetes accounts for around 51% of non-traumatic lower extremity amputations [2].

Various treatment modalities, including aggressive debridement of necrotic tissue, modern dressings with strict control of blood sugars, avoidance of pressure, dressing, and personal care, are being practiced. Particularly in primary care settings, this interdisciplinary approach is linked to the high usage of healthcare resources. Around 15-30% of patients report that the standard therapy is still effective. In addition, skin ulcers often last between eight months and one year, illustrating the severity of the issue for many patients, given the pain, disability, lost wages, and eventual amputation that often occur despite receiving high-quality therapy [3,4]. 

Platelet-rich plasma (PRP) is made up of plasma from the same person with a platelet concentration that is multiple times greater as compared to the baseline level [3]. PRP, which formerly included 0.5x10^11^ platelets per unit, was the term given to the normal platelet concentrate for transfusion in patients with severe thrombocytopenia. In the past ten years, the possibility of using PRP to speed wound healing has been investigated. It might be challenging to identify between various regimens because the majority of these products are sometimes incorrectly referred to as PRP, just like the initial transfusion platelet concentrates [4]. PRP possesses chemotactic and mitogenic characteristics, acting as a growth factor agonist. A high concentration of platelets, as well as all of the clotting and growth factors found in the body, including vascular endothelial growth factor, endothelial growth factor, and insulin-like growth factor, are present [5]. All of these growth elements promote quick wound healing. A ground-breaking discovery in the field of wound healing is PRP [6]. PRP accelerates the healing of wounds by supplying vital growth factors and lowering inflammation [7]. 

The rationale of the study was to compare the efficacy of locally injected PRP in diabetic foot ulcer patients versus conventional dressing. Although it has been proved in previous studies that locally injected PRP is beneficial in the treatment of chronic ulcers [5-9]. Literature regarding the advantages of its use in the treatment of diabetic foot ulcers is not available locally. As people of Pakistan are genetically, geographically, and in terms of a lifestyle different from other populations of the world, the current study has been designed in the local population of Peshawar to compare the efficacy of PRP versus conventional dressing in diabetic foot ulcer patients. The positive outcomes of PRP will be helpful in future recommendations as a treatment modality to decrease morbidity caused by foot ulcers in patients with diabetes mellitus. The current study aimed to compare the efficacy of injected PRP in diabetic foot ulcer treatment in terms of epithelialization of the ulcer as compared to the conventional dressing.

## Materials and methods

A prospective observational study was conducted at the Department of Diabetes and Endocrinology, Lady Reading Hospital, Peshawar, Pakistan, between July 2020 to January 2021. After ethical approval was obtained from the institutional review board (reference #46546), the study was initiated. 

Utilizing the statistical program OPEN EPI, the sample size was determined with a test power of 99% and a confidence interval of 99%.Considering proportions of 81.3% and 42.1% [10] in the study and control groups, respectively, the sample size was calculated to be at least 62 patients in each group. The participants of the study were chosen using a non-randomized, sequential sampling method. The inclusion criteria consisted of the following conditions: i) both male and female patients aged between 35 and 80 years, ii) patients with a proven diagnosis of diabetes mellitus type II, iii) patients that were compliant with their diabetes treatment, and iv) patients with non-infected and non-healing ulcers for more than six weeks.

Patients with ulcers secondary to other causes (e.g., pressure, arterial insufficiency), patients that were non-compliant with their diabetes medication (not regularly taking their medicines), patients who were lost to follow-up, or those taking immunosuppressives like steroids, and those who had multiple co-morbid conditions (e.g., hepatitis C, liver insufficiency, chronic liver disease), patients with multiple or bilateral ulcers, and patients who refused to give informed consent were excluded from the study.

Efficacy of treatment was defined as the ability of injected platelet-rich plasma or a conventional dressing to decrease a single wound grade from a bone line within one month as positively measured on the basis of Wagner's classification [11]. Dyslipidemia was defined by one or more of the following abnormalities: i) total cholesterol >200 mg/dl, ii) hypertriglyceridemia of >150 mg/dl, iii) low density lipoprotein (LDL) cholesterol of >160 mg/dl, iv) high density lipoprotein (HDL) cholesterol of <40 mg/dl. 

The participants were enrolled from the outpatient department. This study included 80 patients with diabetic foot ulcers according to the criteria described. The collected data via a proforma included age, a detailed medical history, duration of diabetes, smoking history, and co-morbid conditions, such as hypertension and dyslipidemia.

Selected patients were divided into two groups of 80 each. Group I (study group) participants received PRP (1 ml/1 cm^2^) around the edges of the wound and the base of the ulcer. 

For PRP preparation, 5 ml of the patient's blood sample was centrifuged. A centrifuge was used to spin the blood sample at high velocity. The platelet-containing supernatant plasma was placed into a different sterile tube (without anticoagulant). To get a platelet concentration, the tube underwent faster centrifugation. A total of three injections were administered, each being two weeks apart, i.e., on day 0, day 14, and day 28. Group II (control group) participants were treated with conventional dressings. Traditional wound care procedure included using gauze, cleaning the ulcer wound with cotton wool, and then drying the wound area to prevent contamination. Ulcer wounds of participants in both groups were inspected on the basis of Wagner's classification on days 0, 14, 28, 90, and 180. Hemoglobin A1c (HbA1c) for all participants was repeated every three months.

Data analysis was done with SPSS version 23.0 (IMB Inc., Armonk, USA). Mean and standard deviation were calculated for age, duration of diabetes, HbA1c, and body mass index (BMI). For qualitative variables like gender/sex, age groups, dyslipidemia, hypertension, smoking history, obesity, and efficacy, frequency and percentages were calculated. A Chi-square test was utilized for comparison of efficacy. A p-value of below 0.05 was considered as statistically significant. 

## Results

A total of 80 patients underwent platelet-rich plasma (PRP) treatment, while 80 patients underwent conventional dressings. The baseline characteristics and biochemical levels are illustrated in Table 1. A decreasing trend of hemoglobin A1c (HbA1c) was observed in both groups.

**Table 1:** Baseline characteristics and biochemical evaluation of study versus control group

Parameters	Study group	Control group	p-value
Age (years)	54.4 ± 8.56	55.7 ± 9.1	0.3543
Body mass index (kg/m^2^)	25.9 ± 5.1	27.2 ± 5.8	0.1342
Hemoglobin A1c at presentation (mmol)	12.3 ± 4.5	11.9 ± 4.2	0.5619
Hemoglobin A1c at 90 days (mmol)	9.7 ± 3.9	9.1 ± 3.7	0.3197
Hemoglobin A1c at 180 days (mmol)	7.3 ± 2.9	7.8 ± 2.5	0.8963
Duration of diabetes (years)	13.4 ± 4.7	12.7 ± 4.3	0.3272

Table 2 illustrates the demographic and clinical profile between the study and the control groups. Dyslipidemia was present in 46.25% of the study group patients and 40% of the controls.

**Table 2:** Comparison of demographic and clinical profiles between study and control groups

Parameters	Study group	Control group	p-value
Gender			
Male	35 (43.8%)	37 (46.3%)	0.751
Female	45 (56.3%)	43 (53.8%)	
Dyslipidemia			
Present	27 (33.8%)	32 (40%)	0.413
Absent	53 (66.25%)	48 (60%)	
Hypertension			
Yes	37 (46.25%)	35 (43.75%)	0.751
No	43 (53.75%)	45 (56.25%)	
Smoking status			
Yes	24 (30%)	27 (33.75%)	0.611
No	56 (70%)	53 (66.25%)	
Obesity			
Yes	21 (26.25%)	19 (23.75%)	0.715
No	59 (73.75%)	61 (76.25%)	

The primary outcome of the study was the efficacy of PRP treatment, as demonstrated in Table 3. Statistically significant reductions in wound were observed in the study group as compared to the control group (p<0.0001).

**Table 3:** Efficacy of platelet-rich plasma (PRP) treatment in the study versus control groups

Reduction in wound	Study group	Control group	p-value
Yes	64 (80%)	37 (46.25%)	< 0.0001
No	16 (20%)	43 (53.75%)	

It was further found that in female patients, compared to the controls, the experimental group's wound healing was markedly better (p<0.0001). Moreover, in patients aged above 55 years, frequently higher rates of wound reduction were observed in the study group as compared to the control group (p<0.0001). Additionally, in patients with normal body mass index (BMI), a significantly higher rate of wound healing was observed (Table 4).

**Table 4:** Stratification of study outcome on the basis of sociodemographic and clinical parameters

Characteristic	Reduction in wound		p-value
	Yes (101)	No (59)	
Gender			
Male			
Study group	27 (77.1%)	8 (22.9%)	0.067
Control group	21 (56.8%)	16 (43.2%)	
Female			
Study group	37 (82.2%)	8 (17.8%)	< 0.0001
Control group	16 (37.2%)	27 (62.8%)	
Age groups			
50-55 years			
Study group	38 (74.5%)	13 (25.5%)	0.081
Control group	21 (56.8%)	16 (43.2%)	
> 55 years			
Study group	26 (89.7%)	3 (10.3%)	< 0.0001
Control group	16 (37.2%)	27 (62.8%)	
Dyslipidemia			
Study group	24 (88.9%)	3 (11.1%)	< 0.0001
Control group	11 (34.4%)	21 (65.6%)	
Hypertension			
Study group	26 (70.3%)	11 (29.7%)	0.005
Control group	13 (37.1%)	22 (62.9%)	
Smoking status			
Study group	16 (66.7%)	8 (33.3%)	0.225
Control group	14 (51.9%)	14 (51.9%)	
Obesity			
Study group	13 (61.9%)	8 (38.1%)	0.796
Control group	11 (57.9%)	8 (42.1%)	
Normal body mass index (BMI)			
Study group	51 (86.4%)	8 (13.6%)	<0.0001
Control group	27 (44.3%)	34 (55.7%)	

## Discussion

Platelet-rich plasma (PRP) therapy methods in chronic wound healing are gaining tremendous popularity because of the successful outcomes. In the present study, the effectiveness of PRP in the management of diabetic foot ulcer was found to be 80%. Our study is in line with the previously published data. For instance, a case series involving 24 patients was published showing signs of wound healing in patients with chronic foot wounds with a reduction in wound size by autologous PRP injection, and the average duration for healing of the ulcer was about eight weeks [12]. Suthar et al. reported efficacy in 17 (70.83%) patients [12]. 

In a recent study, an extremely apparent difference was revealed between the efficacy of the study and control groups (p=0.007) [10]. The findings of our study are comparable with multiple studies discussed below. According to a meta-analysis, PRP therapy enhanced the possibility of healing chronic wounds and decreasing the volume of the ulcer (p<0.01), and reducing the amount of time needed for the process to be accomplished. Additionally, PRP lowered the risk of adverse events (p=0.02) but did not vary from conventional therapy in terms of the likelihood of wound complications or recurrences (p=0.43). There is enough data, according to this meta-analysis, to show a statistically significant advantage of PRP over other treatment modalities [13]. According to another study done in India, the average decrease in the percentage of ulcer area and volume was 91.7% (with SD of 18.4 percent) and 95 percent (with SD of 14 percent), respectively [14]. 

One benefit of PRP is that it can be made readily from patient blood by a straightforward centrifugation procedure, making it a safe, straightforward, and affordable product [15,16]. The amount of growth factors and proteins produced during activation may be regulated by altering the centrifugation settings and the activation process [17].

An unpolluted and unadulterated procedure is required, wherein centrifugation prerequisites are essential to attain an optimum PRP quality along with the quantity of platelets and lymphocytes [18]. This procedure will also help characterize the major elements that have a critical role to play in tissue repair and develop an acceptable preparation for each specific disease or pathology. Platelet gel was used in modest uncontrolled prospective studies in combination with lean split-thickness grafts of skin tissue in addition to the fibrin glue, which acts as a hemostatic sealant that eschews the use of staples or stitches to boost skin-graft take. This is because PRP plays a role in the delivery of mitogenic and chemostatic factors. The investigations included recalcitrant ulcers with a range of etiologies. No negative effects were noticed [19]. In limited diverse case series, negative-pressure wound treatment or light-emitting diodes have also been coupled with a considerable improvement in various healing outcomes [20]. 

Moreover, Scimeca et al. reported that a 49-year-old man who had a deep and non-healing plantar diabetic ulcer for three months had been successfully treated with PRP [21]. Another noteworthy study is a retrospective cohort analysis of 26,599 individuals with diabetic foot ulcers in which 50% of patients who received PRP therapy and 41% of patients who did not receive PRP experienced healing of ulcer with a relative risk of healing with PRP of 1.38 (1.33-1.42) [22].

There are certain limitations of the present study despite its many apparent strengths. For instance, a non-probability convenience sampling technique for participant recruitment rendered the study findings liable to possible selection bias. The authors recommend that large randomized trials should be conducted in order to evaluate the role of PRP in patients with diabetic foot ulcer disease with more certainty. 

PRP is a frequently performed, reasonably priced, and minimally invasive procedure that only takes a small volume of blood from the patient to be drawn many times. If further knowledge about PRP's efficacy, which would result in reliable treatment regimens, is collected, it may play a part in that plan.

## Conclusions

The study concluded that injected platelet-rich plasma was significantly better than conventional dressing in the management of diabetic foot ulcer. More clinical trials are necessary to evaluate the effectiveness of injected platelet-rich plasma to validate the current findings. Furthermore, additional studies of clinical trials in multiple study centers with large samples in Pakistan are needed to truly represent the target population.
